# Insights from structural studies of the cardiovirus 2A protein

**DOI:** 10.1042/BSR20210406

**Published:** 2022-01-20

**Authors:** Neva Caliskan, Chris H. Hill

**Affiliations:** 1 Helmholtz Institute for RNA-based Infection Research (HIRI), Josef-Schneider-Straβe 2/D15, Würzburg 97080, Germany; 2 Medical Faculty, Julius-Maximilians University of Würzburg, Würzburg 97080, Germany; 3 Division of Virology, Department of Pathology, University of Cambridge, Tennis Court Road, Cambridge CB2 1QP, U.K.

**Keywords:** cardiovirus, RNA-binding proteins, translation, virology

## Abstract

Cardioviruses are single-stranded RNA viruses of the family *Picornaviridae.* In addition to being the first example of internal ribosome entry site (IRES) utilization, cardioviruses also employ a series of alternative translation strategies, such as Stop-Go translation and programmed ribosome frameshifting. Here, we focus on cardiovirus 2A protein, which is not only a primary virulence factor, but also exerts crucial regulatory functions during translation, including activation of viral ribosome frameshifting and inhibition of host cap-dependent translation. Only recently, biochemical and structural studies have allowed us to close the gaps in our knowledge of how cardiovirus 2A is able to act in diverse translation-related processes as a novel RNA-binding protein. This review will summarize these findings, which ultimately may lead to the discovery of other RNA-mediated gene expression strategies across a broad range of RNA viruses.

## Introducing the cardioviruses

Viruses are obligate intracellular pathogens that depend on the host translation machinery to translate their own genome and replicate within the cell [1]. In the case of picornaviruses, the single-stranded positive-sense RNA genome serves as both the genetic blueprint for replication and as a messenger RNA (mRNA) template for translation of all viral protein components [2]. Once the viral RNA molecule enters the host cell, it must hijack host ribosomes to translate itself whilst escaping from the intracellular immune surveillance systems, which act to restrict viral spread through inhibiting translation and viral replication (reviewed in [3–6]). For that, viruses have evolved sophisticated non-canonical gene expression mechanisms to allow efficient production of viral proteins [7,8]. One such example is a group of phenomena collectively termed ‘recoding’, which occur on specific coding sequences during the elongation step of translation. Recoding either alters the interpretation of individual codons (e.g. stop-codon readthrough), or the entire meaning of the code by moving to an alternative reading frame on the mRNA (e.g. frameshifting) [9–11].

Cardioviruses comprise a diverse group of viruses within the family *Picornaviridae* [12]. They have been isolated from a variety of mammalian, avian and invertebrate species and cause encephalitis, myocarditis and enteric disease in rodents, swine and humans [13,14]. The genus cardiovirus is further divided into six species [12]. The archetype of *Cardiovirus A* is the encephalomyocarditis virus (EMCV), a well-established model for studying non-canonical translation [15]. The second species, *Cardiovirus B* or *Theilovirus*, includes rat theilovirus (RTV), Vilyuisk human encephalomyelitis virus (VHEV) and Theiler’s murine encephalomyelitis virus (TMEV), all of which are genetically divergent from *Cardiovirus A* [16]. Within this group, TMEV is the most extensively characterized virus and has been employed as a model to study virus-mediated demyelination and multiple sclerosis [17]. Human Saffold virus (SAFV) was previously a member of *Cardiovirus B* before re-classification as new species *Cardiovirus D* in 2019 [13]. To date, *Cardiovirus C*, *E* and *F* each comprise only one or two isolates [12,18].

**Figure 1:**
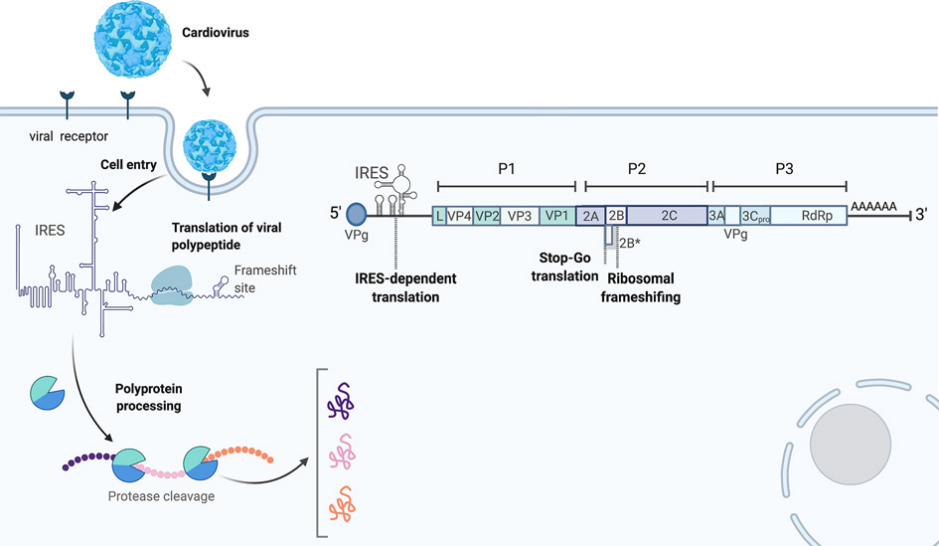
Representation of cardiovirus cell entry, translation and genome organization Upon cell entry, viral (+) single-stranded RNA is translated in the cytoplasm to produce three large precursor proteins (P1, P2 and P3), which are cleaved by proteases to yield functional proteins. Viral genomic RNA contains VPg and IRES at its 5′ untranslated region for efficient translation. The coding region of the viral RNA also contains Stop-Go and frameshifting sites at the 2A–2B junction, which mediate non-canonical translation events (created with BioRender).

Like other picornaviruses, all viral processes following viral entry take place in the cytoplasm, including replication, translation and viral assembly [12,15,19]. The life cycle of cardioviruses is summarized in Figure 1. Viral entry through the cognate receptor is followed by uncoating and the release of the viral genetic material [20,21]. The viral positive-sense RNA is transcribed into the complementary negative strand, which then serves as a template for the synthesis of the progeny virus RNA (not shown in figure) [12,15]. The positive-sense RNA is also used as the mRNA template for translation of the structural and replicative proteins. The single-stranded ∼8 kilobases (kb) positive-sense RNA genome of the cardiovirus consists of a single open reading frame (ORF) comprising three regions: P1 which encodes structural proteins, and P2 and P3, which encode non-structural proteins [22]. The product of translation is a polyprotein (L-1ABCD-2ABC-3ABCD, ∼2200 amino acids), which is then processed into ∼12 protein products, mainly by the virally encoded 3C polypeptide, a chymotrypsin-like cysteine protease [23]. Together VP1, VP2, VP3 and VP4 form the viral capsid [24,25]; 2B is a viroporin that alters membrane integrity and permeability [26,27]; 2C has ATPase and putative helicase activities [28]; 3A is membrane-associated and recruits phosphatidylinositol-4 kinase IIIα to replication organelles [29]; 3B is VPg (viral protein genome-linked) [30] and 3D is the RNA-dependent RNA-polymerase [31]. L and 2A are the primary virulence factors. Their amino acid sequences are amongst the most divergent between cardiovirus isolates, and the molecular basis for their activity is still not completely understood [15,32].

## Unusual gene expression strategies employed by the cardioviruses

Despite sharing a similar genomic organization to other picornaviruses, cardioviruses possess several unique features that facilitate key regulatory events during genome translation, thus allowing the virus to regulate its own replication and interfere with host cellular processes. Similar to other picornaviruses, the polyprotein encoding ORF is flanked at both ends by lengthy untranslated regions (UTRs) with significant secondary and tertiary structures [22,33]. The 3′ end of the viral RNA is polyadenylated, thus resembling a characteristic modification of 3′ eukaryotic protein-coding mRNAs [22,23]. However, unlike most eukaryotic mRNAs, instead of a 5′ 7-methyl-guanosine cap structure, the 5′ end of the cardiovirus genome is covalently attached to VPg protein through a phosphotyrosyl linkage. Presence of VPg is essential for the synthesis of the negative strand for viral replication [34]. In some picornaviruses such as in poliovirus (PV), the first ⁓100 nucleotides of the 5′ UTR contain a peculiar cloverleaf RNA motif, which enhances translation, and is important for organizing viral and cellular proteins involved in the RNA synthesis [35–38]. This region interacts with many proteins including viral polypeptides 3CD and the host poly(C)-binding protein (PCBP) [35]. Furthermore, protein–protein interactions between the cloverleaf-3CD-PCBP and another host protein, the poly(A)-binding protein (PABP) might form long-range interactions, bringing 5′ and 3′ UTRs of the genome into close proximity, and thereby facilitating re-attachment of ribosomes to the 5′ end of the genome following a round of translation [39]. Surprisingly, in cardioviruses (and aphthoviruses like the Foot-and-mouth-disease virus, FMDV) the cloverleaf motif is absent [33,40]. Instead, their 5′ UTRs contain a poly(C) tract of variable length (60–350 nucleotides), followed by pseudoknots of unknown function [41]. Truncation of this region reveals that the virus remains viable and induces immune responses in the host. However, variants with short poly(C) tracts display diminished pathogenicity, making them promising vaccine candidates [42,43]. Exactly how poly(C) tract interactions work at the molecular level, and precisely which viral and host factors are involved warrant further studies.

Other conserved RNA structures both at the 5′ UTR and within the coding region of the viral genome are also important for regulating viral gene expression. A region of ⁓450 nucleotides within the 5′ UTR comprises a type II internal ribosome entry site (IRES), which directs cap-independent internal initiation of protein synthesis [44,45]. In cardioviruses (e.g. EMCV) and aphthoviruses (e.g. FMDV), these elements only share 50% sequence identity [45], yet display similar secondary structures. Picornaviral IRESs also show intriguing functional differences in cell-free translation assays. For instance, it was observed that the IRES found in enteroviruses (e.g. PV) has low activity in rabbit reticulocyte lysate (RRL) but is stimulated by the addition of HeLa cell extracts [46]. Conversely, type II IRES elements of cardioviruses work efficiently in RRL, pointing to differences in factor requirements and the involvement of species-specific IRES trans-acting factors (ITAFs) for efficient translation initiation (reviewed in [41,47]). Well-characterized RNA-binding proteins known to enhance IRES function include (but are not limited to) LA autoantigen [48], polypyrimidine tract binding protein (PTB) [49] and poly(C)-binding protein (PCBP2) [50,51]. The role of these RNA-binding proteins could be the stabilization or modification of the IRES RNA structure, thus allowing conserved parts of the RNA to interact with the translation apparatus during translation initiation [48,52,53].

Although the 5′ UTR of cardioviruses has been a hotspot of research, other translational regulatory regions within the coding sequences are also crucial for viral pathogenicity. The coding region of the cardioviruses contains a conserved RNA element that directs programmed–1 ribosomal frameshifting (PRF) at the junction of 2A–2B genes [54]. Ribosome profiling of infected cells has revealed the efficiency of this PRF event (75–84%) is amongst the highest known in any virus [55,56]. Frameshifting occurs 11–12 codon into the 2B gene, leading to the production of the 2B* *trans*-frame product. In EMCV this is a 128-amino acid protein, whereas in TMEV it is a short 14–15-amino acid peptide [54]. 2B encodes a viroporin, which increases cell permeability and thereby can induce viral release and host cell apoptosis [57]. On the other hand, 2B* has no known function and the main role of frameshifting is thought to be the down-regulation of the other proteins encoded downstream of the frameshift site [58]. Furthermore, at the junction of 2A–2B, the virus also employs a Stop-Go or ribosome skipping event. Unlike frameshifting, Stop-Go translation is not driven directly by RNA elements, but instead, an interaction of the nascent peptide with the elongating ribosome [59]. This leads to the failure of peptide bond formation between Gly-Pro within a conserved Asn-Pro-Gly-Pro motif, and the peptide upstream of the second proline is released [60]. This event is redundant in some cardioviruses due to the presence of the 3C protease cleavage site at the start of the 2B [54]. Overall, the multitude of alternative translation events occurring at the 2A–2B junction points to the importance of this region for the viral life cycle.

## Protein-mediated frameshifting as an emerging paradigm in viral gene expression

Before we discuss protein-mediated frameshifting, it is useful to briefly consider the general principles of conventional PRF, applicable to hundreds of RNA viruses. Frameshifting ensures the production of proteins in optimal ratios for efficient virus assembly and enables viruses to expand their coding capacity through the utilization of overlapping ORFs [61–63]. Typically, PRF occurs on heptanucleotide motifs preceding strong RNA secondary structures. In canonical PRF, elongating ribosomes pause over a ‘slippery sequence’ of the form X_XXY_YYZ (where XXX is any three identical nucleotides or other triplet such as GGU, YYY is AAA or UUU, and Z is any nucleotide except G) when they encounter a structured RNA ‘stimulatory element’ 5–9 nucleotides downstream. During this time, a −1 frameshift may occur if codon–anticodon re-coupling takes place over the X_XXY_YYZ sequence: the homopolymeric stretches allow the tRNA in the P-site tRNA to slip from XXY to XXX, and the tRNA in the A-site to slip from YYZ to YYY [61,63,64]. A variety of stem-loops and pseudoknots are known to induce frameshifting [63]. To date, there is evidence in bacterial systems that the RNA structure impedes the progression of the ribosomes in the canonical reading frame and frameshifting can occur during EF-G mediated translocation of tRNAs [65–68]. The downstream RNA structure hinders the back rotation of the small subunit, thus trapping the ribosome in a chimeric rotated or hyper-rotated state [65,68,69]. Moving to the −1 position re-positions the structure within the mRNA entry tunnel and allows for a more efficient unfolding of the RNA by the intrinsic helicase of the ribosome [65]. However, the stability of the RNA structure by itself does not define the levels of frameshifting. For instance, in *Escherichia coli* a simple hairpin can lead to 50% frameshifting on the dnaX coding sequence and is largely defined by the thermodynamics of codon–anticodon base pairing rather than the kinetics of RNA unfolding [70,71]. Frameshifting usually occurs at a constant efficiency, which provides a fixed ratio of upstream (0 frame) and downstream (−1 frame) gene products. Many RNA viruses exploit this to precisely control levels of structural (0 frame) and replicative (−1 frame) proteins. Small perturbations in PRF efficiency can therefore impact virulence by altering the stoichiometry of viral proteins [72,73].

Recently, it was discovered that arteriviruses and cardioviruses utilize a new mechanism of PRF in which the stimulatory element is not limited to mRNA structures, but also involves an RNA-binding protein [55,74,75]. In the porcine respiratory reproductive syndrome virus (PRRSV, family *Arteriviridae*), a −2 frameshift event occurs during translation of the ORF1a on a G_GUU_UUU slippery sequence. Unlike typical frameshift motifs, instead of the usual downstream structured RNA element, a C-rich sequence (CCCANCUCC) is present [76]. Frameshifting relies on interactions amongst this C-rich RNA element, host PCBP and the viral nsp1β protein [74]. In cardioviruses, the viral 2A protein acts as an essential *trans-*activator for −1 frameshifting [54,55,58]. The frameshift signal comprises the slippery sequence G_GUU_UUX and an RNA–protein complex formed between a downstream stem-loop and the 2A protein [55]. This unique mechanism allows for temporal control of gene expression as the efficiency of −1 frameshifting is linked to 2A concentration, which increases with time throughout the infection cycle [55]. In this way, 2A-induced frameshifting leads to down-regulation of replicative proteins at later stages of infection. These examples of ‘protein-mediated frameshifting’ represent an exquisite illustration of the complexity of viral gene regulatory mechanisms, ensuring appropriate levels of viral proteins are produced at different stages of the viral life cycle by partitioning translating ribosomes into different reading frames [77].

## Cardiovirus 2A as an RNA-binding protein and frameshift stimulator

2A proteins are highly divergent between *Cardiovirus* species, with only ∼14% pairwise amino acid sequence identity (see Figure 2 in [56]). 2A is a small, basic protein (∼14–17 kDa) released from the viral polyprotein by 3C-mediated proteolytic cleavage at the N-terminus and Stop-Go peptide release at a C-terminal 18-amino acid consensus sequence [78,79]. Many other picornaviruses have identically named ‘2A’ proteins that are chymotrypsin-like proteases involved in polyprotein processing [80]. However, cardiovirus 2A has no homology to any of these and displays no protease activity [80–82]. In infected cells, a significant proportion of 2A is nucleolar [30], and a mutational analysis identified a putative nuclear localization sequence (NLS, aa 91–102) similar to those found in yeast ribosomal proteins [78].

**Figure 2:**
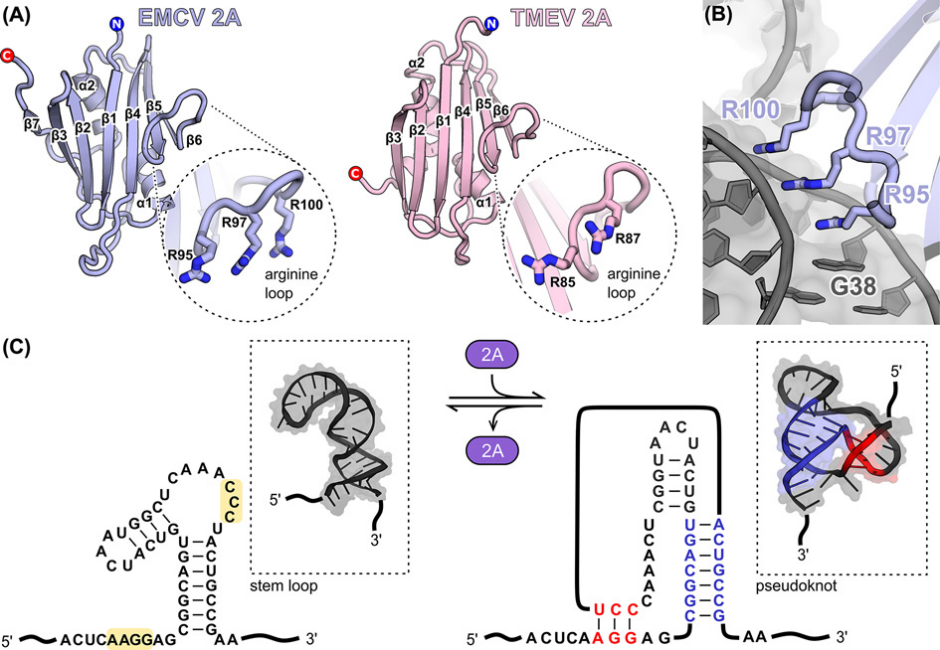
Cardiovirus 2A as an RNA-binding protein and frameshift stimulator (**A**) X-ray crystal structures of the 2A protein from EMCV (left) and TMEV (right). A zoomed-in view of the conserved ‘arginine loop’ is shown. (**B**) Cryo-EM structure of EMCV 2A bound to the *E. coli* small ribosome subunit. The arginine loop is inserted into a helical junction in the 16S rRNA. The interaction involves multiple electrostatic contacts with the ribose phosphate backbone and hydrophobic stacking of the guanidinium groups against each other and an exposed base. (**C**) The EMCV RNA stimulatory element is predicted to adopt both stem-loop (left) and pseudoknot (right) conformations. Conserved sequence elements important for the interaction are highlighted (yellow, left). Recognition of this element by 2A requires both the main stem (blue, right) and additional base-pairing interactions between conserved elements (red, right).

Several recent studies suggest that the primary function of 2A in cells is to stimulate PRF. It was originally identified as a necessary *trans-*activator following observations that cardiovirus PRF occured during infection but could not be recapitulated using *in vitro* translation systems [54]. Subsequent work demonstrated that 2A has RNA-binding activity, and that the addition of recombinant 2A rescues frameshifting *in vitro*. It was proposed that 2A acts by binding to a stem-loop downstream from the G_GUU_UUU slippery sequence [55,58,75]. This model helped to explain another puzzling observation: in cardioviruses, the spacing between the slippery sequence and the stem-loop is 13 nt, significantly longer than the 5–9 nt usually observed, seemingly too long to position the P-site of the ribosome over the slippery sequence during a pause. 2A was thought to fill this gap, acting as a ‘bridge’ between the stem-loop and the ribosome. However, until recently, the lack of structural information made it difficult to understand the molecular basis for these activities.

We have recently reported X-ray crystal structures of 2A protein from both EMCV [83] and TMEV [56]. Strikingly, despite the low sequence identity amongst *Cardiovirus* orthologs, they adopt a common architecture, with no structural homology to any other protein. This β_3_αβ_3_αβ ‘*β*-shell’ fold comprises a six- or seven-stranded antiparallel β-sheet, packed against two α-helices (Figure 2A). Notably, several previously described truncation mutants lack substantial portions of secondary structure and expose elements of the 2A protein hydrophobic core. This would severely disrupt the folding of the protein and the results obtained with these mutants should be interpreted with caution [32,78,84]. In both EMCV and TMEV 2A, the outer convex surface of the β-sheet is enriched in lysine, histidine and arginine residues, conferring a strong positive electrostatic surface potential. One of the most conserved elements is the flexible ‘arginine loop’, located between β5 and β6 (Figure 2A). This loop is essential for both RNA binding and frameshifting [55,58,75], and the cryo-EM structure of EMCV 2A bound to 70S ribosomes reveals that it is central to the RNA-binding surface [83], forming electrostatic contacts with the ribose phosphate backbone and hydrophobic stacking interactions with exposed bases (Figure 2B). Beyond this loop, the RNA-binding surface observed for EMCV 2A is not well-conserved in TMEV 2A. Nevertheless, mutation of surface-exposed basic residues (e.g. R85, R87, K24 or R28) inhibits TMEV 2A function, consistent with an electrostatic RNA-recognition mechanism involving positively charged residues on the central β-sheet [56]. Mutation of buried arginine residues did not have an effect [75].

For both TMEV and EMCV, steady-state binding experiments between 2A proteins and cognate RNA stimulatory elements have provided insights into RNA recognition [56,83]. 2A binding is high affinity (∼450 nM), exergonic, and occurs with 1:1 stoichiometry [56,83]. Interestingly, short RNAs comprising just the predicted stem-loop were not bound by 2A, even at high concentrations (∼32 μM). In both viruses, for binding to occur, it was also necessary to include a conserved GG motif in the region immediately 5′ to the stem-loop, suggesting that 2A interacts both with the stem-loop and the 5′ side of the predicted stem-loop. The involvement of additional nucleotides 5′ to the predicted stem-loop would also decrease the distance between the slippery sequence and stimulatory element, bringing it to ∼9 nt, similar to other protein-independent PRF signals [56,83]. Further insights were gained by mutagenesis experiments. Disruption of either a conserved CCC triplet in the loop, or the 5′ GG element, inhibited 2A binding. Strikingly, in EMCV, a C→G loop mutation that inhibited 2A binding could be rescued by a 5′ G→C mutation, which suggested that base pairing between these nucleotides, and the resultant formation of an alternate pseudoknot-like conformation is essential for 2A recognition [83] (Figure 2C). In all these experiments, a loss of 2A binding to the stimulatory RNA element was intimately linked to a failure to stimulate frameshifting, underlining that the RNA-binding capabilities of 2A are essential to its primary function. This was further explored at the single-molecule level, and experiments with optical tweezers showed that 2A binding stabilized the EMCV RNA stimulatory element, increasing the required unwinding force from ∼10 to ∼27 pN [83]. This would exceed the ∼20 pN unwinding force exerted by the ribosome during elongation [85], thereby providing a mechanistic explanation for ribosome pausing and frameshifting.

These detailed interaction studies support the idea that 2A selectively recognizes a particular RNA conformation of the EMCV and TMEV RNAs, and by stabilizing the RNA enough to induce a ribosomal pause, likely acts as a switch to induce frameshifting (Figure 2C). In this way, control of viral gene expression is temporally regulated by accumulation of 2A during infection. Still, exactly how 2A specifically interacts with these RNAs awaits further investigation.

## Cardiovirus 2A as a multifunctional virulence factor

Besides its primary role in stimulating frameshifting, 2A causes a variety of pathological effects in the host cell. Perhaps most notably, it contributes to translational shut-off [86,87]. Many picornaviruses do this by proteolytically cleaving eukaryotic initiation factor 4G (eIF4G), which prevents assembly of the eukaryotic initiation factor 4F complex (eIF4F) and thereby inhibits cap-dependent initiation on host mRNAs (reviewed in [41,88]). Translation of the viral RNA is unaffected by this, as initiation occurs via a type II IRES that requires all initiation factors except eukaryotic initiation factor 1, 1A and intact eIF4F [89,90]. However, in cardiovirus infection eIF4G is not cleaved [81,82], necessitating an alternative explanation. In normal translation, a YxxxxLΦ motif in eIF4G mediates binding to eukaryotic initiation factor 4E, thereby forming eIF4F and promoting cap-dependent initiation. Host 4E-binding proteins (4E-BPs) are negative regulators that also contain a YxxxxLΦ motif, thus competing for and sequestering eIF4E [91]. The activity of 4E-BPs is further regulated by inhibitory phosphorylation (reviewed in [92]).

**Figure 3:**
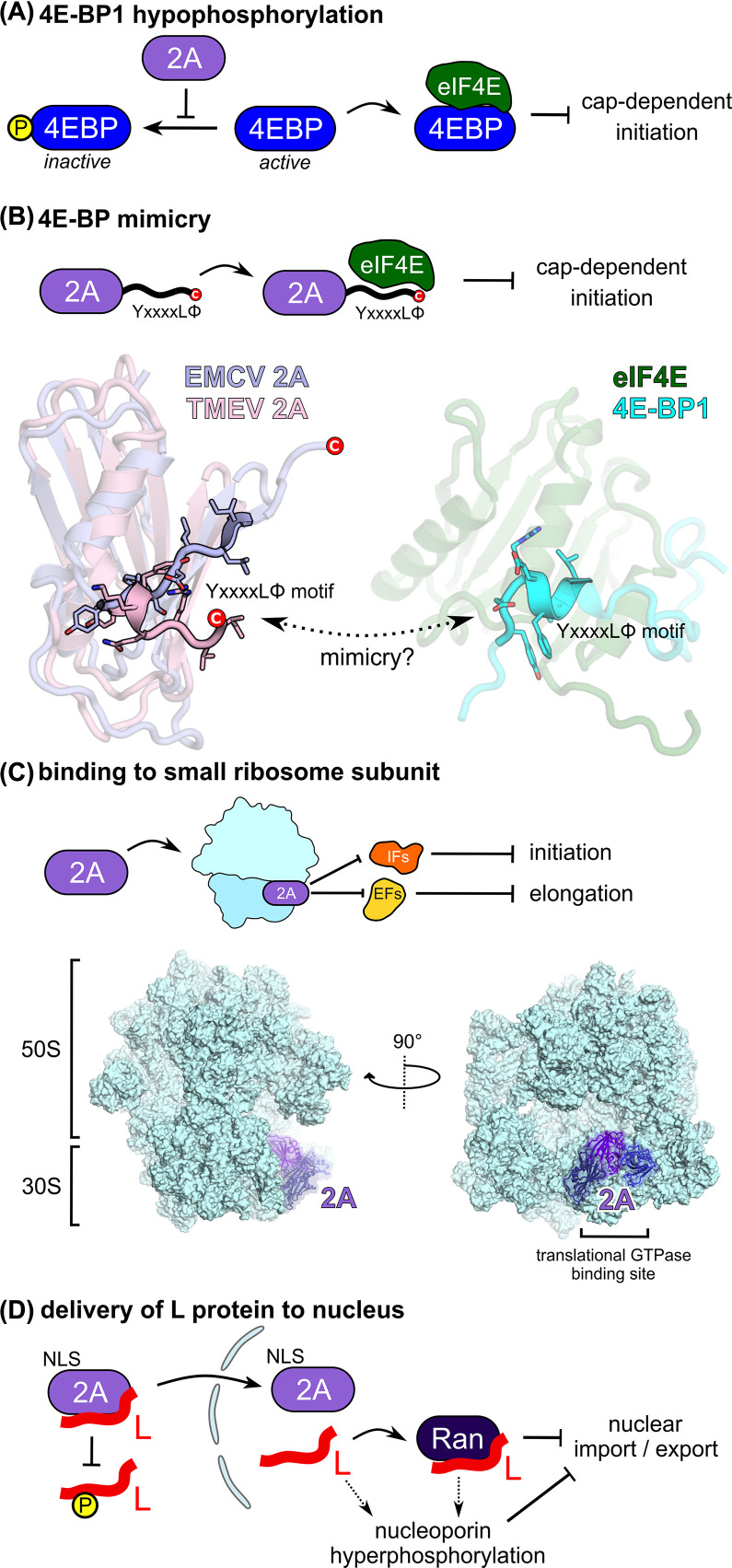
Cardiovirus 2A as a multifunctional virulence factor (**A**) Proposed mechanism for inhibition of cap-dependent translation by mimicry of eIF4E-binding protein (4E-BP) and sequestration of eukaryotic initiation factor 4E (eIF4E). However, the C-terminal YxxxxLΦ motif is not structurally conserved between 4E-BP1 and 2A. (**B**) Proposed mechanism for inhibition of cap-dependent translation by 2A preventing phosphorylation of 4E-BP. (**C**) Alternative model for translational pathology caused by 2A binding to the small ribosome subunit and competing with translational GTPases. (**D**) Proposed role for 2A in delivering L protein to the cell nucleus, where it binds to Ran and causes inhibition of nucleocytoplasmic trafficking.

There are two models for how 2A protein may affect cap-dependent translation by modulating the availability or activity of eIF4E. Firstly, activatory hypophosphorylation of 4E-BP1 was observed during EMCV infection and linked to host–cell shut off [93] (Figure 3A). This was subsequently shown to be 2A-dependent in an experimental model using infected BHK-21 cells [84]. However, another study did not observe a decrease in 4E-BP1 phosphorylation when using HeLa or L cells [78]. These authors propose an alternative model, in which a conserved C-terminal YxxxxLΦ motif in 2A mimics that of 4E-BP1, directly binding and sequestering eIF4E in a functionally analogous way (Figure 3B). If this mimicry model were correct, one would expect this C-terminal motif in 2A to be highly structurally conserved, and to resemble that of the YxxxxLΦ motif in the 4E-BP1–eIF4E complex structure [94]. Our recent structures of EMCV and TMEV 2A reveal that, surprisingly, the C-terminus is highly divergent and this YxxxxLΦ motif is not structurally conserved. In EMCV 2A, it is present in β7, whereas in TMEV 2A it is partially buried and present in a kinked α2 helix (Figure 3B) [56,83]. Neither of these conformations resemble that of the equivalent motif in the 4E-BP1–eIF4E complex structure, suggesting that, without a significant conformational change, this element is unlikely to be the primary determinant of the 2A–eIF4E interaction. Furthermore, it is unclear how relevant the 2A–eIF4E interaction is to host cell shut-off, as viruses harboring mutations in the putative YxxxxLΦ motif were still able to inhibit cap-dependent translation of host mRNAs, despite losing the ability to bind eIF4E [78].

EMCV 2A protein has also been shown to interact directly with 40S subunits in infected cells [95] and *in vitro* with very high apparent affinities (∼10 nM) [56]. This may be relevant to the inhibition of host cell translation. Our recent cryo-EM structure of EMCV 2A in complex with initiated 70S ribosomes shows that several copies of 2A use the arginine loop to bind directly to a conserved patch of 16S rRNA on the small subunit that also acts as the binding site for translational GTPases (e.g. EF-G/eEF2, EF-Tu/eEF1A) [56] (Figure 3C). Such competition would likely have an inhibitory effect on both initiation and elongation, although it is unclear how this would be selective for host and not viral translation. However, tight binding to ribosomal RNA may help to explain the nucleolar localization of 2A in infected cells [78]. Early work demonstrating co-fractionation of 2A with 40S identified that a small proportion of 2A remained tightly bound to 40S despite high-salt washes (750 mM) [95]. Given the potential ability of 2A to bind to multiple sites, it is an intriguing possibility that nucleolar 2A may be bound to immature ribosomal RNA during biogenesis. The existence of several populations of 2A-40S with different salt sensitivity implies that there may be several modes of interaction [95]. The functional consequences of this on host vs. viral translation would depend on how and where 2A was incorporated, as well as 2A concentration during infection.

Disruption of nucleocytoplasmic trafficking is another hallmark of host cell shut-off in cardiovirus infection [96]. The leader (L) protein is predominantly responsible for this. It binds to the Ran GTPase and triggers a cascade of reactions resulting in the hyperphosphorylation of nucleoporins, and the resultant inhibition of nuclear import and export [97–99]. This process is also dependent on L phosphorylation by cellular kinases [100] (e.g. AMPK, CK2 and SYK), although this is not required for binding to Ran. 2A directly binds to L in a 1:1 ratio with moderate affinity (∼1.5 μM) and has been proposed to act as a trafficking adapter, facilitating delivery of L to the cell nucleus, where L dissociates and instead binds to Ran (∼3 nM affinity) (Figure 3D) [32]. Truncation experiments indicate that the L interaction surface on EMCV 2A is within the first 50 N-terminal amino acids [32]. This places it within the first three beta strands of the central sheet (Figure 2A), leaving the ‘arginine loop’ free to act as an NLS.

Finally, 2A has been implicated in the inhibition of apoptosis during EMCV infection [101]. Virus release is normally cytolytic (necrotic) [102]. However, in a Δ2A virus, BHK-21 cells were observed to undergo cell death by apoptosis, with evidence of caspase-3 activation [101]. It is unclear to what extent this is a direct effect, as this virus may also have defects in polyprotein processing and frameshifting, potentially altering the stoichiometry of other viral proteins. Given that the L protein is a strong anti-apoptotic factor [102], another possibility is that the absence of 2A leads to a mislocalization of L protein, thereby attenuating its effects.

## Concluding remarks: the arginine loop as a nexus for 2A activity

The arginine loop (Figure 2A) was first identified as a functional NLS, sufficient to drive nuclear localization when fused to an eGFP reporter [78]. This is also the most conserved part of the entire 2A sequence. Subsequent structural and biochemical work demonstrated that it comprises an essential part of the 2A RNA-binding surface, and is indispensable for frameshift stimulation and ribosome binding (Figure 2B) [55,75]. The central role of this loop in so many viral activities is surprising and makes mutagenesis studies problematic to interpret because mutants will display composite phenotypes derived from an inability to activate frameshifting, bind to ribosomes, traffic to the nucleolus or deliver L protein to the nucleus. Importantly, it also implies that per 2A molecule, these events are mutually exclusive. This raises further questions about how the relative nuclear vs. cytoplasmic pools of 2A are maintained, and whether 2A functions differently at the early and late stages of infection. Although the recent structures provide a detailed atomic framework for the interpretation of many virological observations, further work will be required to carefully dissect these activities in time and space.

## Abbreviations

eIF4F: eukaryotic initiation factor 4F complex
eIF4G: eukaryotic initiation factor 4G
EMCV: encephalomyocarditis virus
FMDV: foot-and-mouth-disease virus
IRES: internal ribosome entry site
ITAF: IRES trans-acting factor
mRNA: messenger RNA
NLS: nuclear localization sequence
ORF: open reading frame
PCBP: poly(C)-binding protein
PRF: –1 programmed ribosomal frameshifting
PV: poliovirus
RRL: rabbit reticulocyte lysate
TMEV: Theiler’s murine encephalomyelitis virus
UTR: untranslated region
VPg: viral protein genome-linked
4E-BP: 4E-binding protein
